# Gut Microbiota and Gestational Diabetes Mellitus: A Review of Host-Gut Microbiota Interactions and Their Therapeutic Potential

**DOI:** 10.3389/fcimb.2020.00188

**Published:** 2020-05-15

**Authors:** Zubaidah Hasain, Norfilza Mohd Mokhtar, Nor Azmi Kamaruddin, Nor Azlin Mohamed Ismail, Nurul Huda Razalli, Justin Vijay Gnanou, Raja Affendi Raja Ali

**Affiliations:** ^1^Department of Physiology, Faculty of Medicine, Universiti Kebangsaan Malaysia, Cheras, Malaysia; ^2^Faculty of Medicine, National Defence University of Malaysia, Kuala Lumpur, Malaysia; ^3^GUT Research Group, Faculty of Medicine, Universiti Kebangsaan Malaysia, Kuala Lumpur, Malaysia; ^4^Endocrine Unit, Department of Medicine, Faculty of Medicine, Universiti Kebangsaan Malaysia, Cheras, Malaysia; ^5^Department of Obstetrics & Gynecology, Faculty of Medicine, Universiti Kebangsaan Malaysia, Kuala Lumpur, Malaysia; ^6^Dietetic Program, Faculty of Health Sciences, Universiti Kebangsaan Malaysia, Kuala Lumpur, Malaysia; ^7^School of Medicine, International Medical University, Bukit Jalil, Malaysia; ^8^Gastroenterology Unit, Department of Medicine, Faculty of Medicine, Universiti Kebangsaan Malaysia, Cheras, Malaysia

**Keywords:** gut microbiota, gestational diabetes, probiotics, host microbial interactions, short-chain fatty acids

## Abstract

Gestational diabetes mellitus (GDM) is defined as impaired glucose tolerance recognized during pregnancy. GDM is associated with metabolic disorder phenotypes, such as obesity, low-grade inflammation, and insulin resistance. Following delivery, nearly half of the women with a history of GDM have persistent postpartum glucose intolerance and an increased risk of developing type 2 diabetes mellitus (T2DM), as much as 7-fold. The alarming upward trend may worsen the socioeconomic burden worldwide. Accumulating evidence strongly associates gut microbiota dysbiosis in women with GDM, similar to the T2DM profile. Several metagenomics studies have shown gut microbiota, such as Ruminococcaceae, *Parabacteroides distasonis*, and *Prevotella*, were enriched in women with GDM. These microbiota populations are associated with metabolic pathways for carbohydrate metabolism and insulin signaling, suggesting a potential “gut microbiota signature” in women with GDM. Furthermore, elevated expression of serum zonulin, a marker of gut epithelial permeability, during early pregnancy in women with GDM indicates a possible link between gut microbiota and GDM. Nevertheless, few studies have revealed discrepant results, and the interplay between gut microbiota dysbiosis and host metabolism in women with GDM is yet to be elucidated. Lifestyle modification and pharmacological treatment with metformin showed evidence of modulation of gut microbiota and proved to be beneficial to maintain glucose homeostasis in T2DM. Nonetheless, post-GDM women have poor compliance toward lifestyle modification after delivery, and metformin treatment remains controversial as a T2DM preventive strategy. We hypothesized modulation of the composition of gut microbiota with probiotics supplementation may reverse postpartum glucose intolerance in post-GDM women. In this review, we addressed gut microbiota dysbiosis and the possible mechanistic links between the host and gut microbiota in women with GDM. Furthermore, this review highlights the potential therapeutic use of probiotics in post-GDM women as a T2DM preventive strategy.

## Introduction

Gut microbiota refers to the collection of microorganisms present within the digestive tract. Approximately 100 trillion gut microbiota, including bacteria, archaea, viruses, and eukaryotic microbes reside in the human gut, mainly at the distal colon (Bäckhed et al., 2005; Qin et al., 2010). Traditionally, conventional culture techniques were used to map the gut microbiota. However, this technique is outdated, as it may require a consistent anaerobic environment for certain bacterial species and has limited robustness. The advent of culture-independent next-generation sequencing (NGS) platforms has enabled high-throughput molecular sequencing of gut microbiota to explore the complex host-gut microbial interactions efficiently. The two commonly used metagenomics approaches to analyze the gut microbiota are 16S sequencing and shotgun metagenomics sequencing. The 16S sequencing approach is preferable for its affordability and focus, as well as easy bioinformatic analysis, compared to shotgun metagenomics sequencing. The 16S sequencing approach targets only the hypervariable regions of the 16S ribosomal RNA gene (V1 to V9). The Metagenomics of the Human Intestinal Tract (MetaHIT) consortium recommends the V4 region as a gold standard for gut microbiota profiling (Qin et al., 2010). However, 16S sequencing has limited taxonomical and functional resolutions. In contrast, shotgun metagenomics sequencing refers to a massive parallel sequencing of DNA samples that offers better resolution and more specific taxonomic and functional classifications of sequences compared to 16S sequencing. Nevertheless, bioinformatic analysis for shotgun metagenomics is challenging and arduous, because shotgun metagenomics generates a large amount of data (Franzosa et al., 2014; Jovel et al., 2016).

The ability to characterize gut microbiota via NGS platforms has improved our understanding of the link between gut microbiota and human health as well as its association with multiple diseases, primarily when compositional perturbations of the microbiota occurs. In general, gut microbiota maintains host interaction through digestion, metabolism, extraction of nutrients, synthesis of vitamins, protection against pathogens, and systemic immunomodulation (Qin et al., 2010; Grenham et al., 2011; Flint et al., 2012; Huttenhower et al., 2012). In healthy adults, the predominant gut microbiota compositions are Bacteroidetes and Firmicutes phyla (Lloyd-Price et al., 2016). Human gut microbiota is subclassified into two major groups: commensal symbionts and pathobionts. Commensal symbionts are usually predominant in a healthy population and confer a symbiotic relationship with the host (Hornef, 2015). An example of commensal symbionts is butyrate-producing bacteria, such as *Faecalibacterium prausnitzii*. It is a member of the Firmicutes phylum, exerts anti-inflammatory action and takes part in energy metabolism (Miquel et al., 2014; Tilg and Moschen, 2014). Pathobionts are a group of bacteria that trigger a pathogenic inflammatory response and induce harmful effects on the host when elevated (Hornef, 2015). Proteobacteria is a Gram-negative pathobiont, which has been associated with inflammation in patients with T2DM (Salguero et al., 2019).

Altered normal gut microbiota composition or dysbiosis refers to an imbalance between commensal symbionts and pathobionts (Taddei et al., 2018). Previous studies have reported a positive association between gut microbiota dysbiosis and disorders such as inflammatory bowel disease, obesity, and T2DM (Le Chatelier et al., 2013; Han and Lin, 2014; Ibrahim et al., 2017; Lee et al., 2017; Ananthakrishnan et al., 2018). In a review by Han and Lin (2014), most of the studies that investigated the association between gut microbiota and T2DM observed evidence of gut microbiota dysbiosis, elevation of pathobionts and reduction of beneficial butyrate-producing bacteria. For example, a study by Larsen et al. (2010) compared gut microbiota composition between healthy adults and adults with T2DM. They identified pathobionts, such as Bacteroidetes and Proteobacteria, that were highly enriched and linearly associated with the plasma glucose level, whereas Firmicutes and *Clostridia* were significantly reduced in men with T2DM compared to the healthy controls (Larsen et al., 2010). Furthermore, Qin et al. (2012) have discovered adults with T2DM have significantly depleted levels of butyrate-producing bacteria, such as *Roseburia intestinalis* and *F. prausnitzii*, and an increased abundance of pathobionts, such as *Bacteroides caccae, Clostridiales, Escherichia coli*, and *Desulfovibrio*. However, some of the studies have shown conflicting results, where the abundance of Firmicutes and *Lactobacillus* was noted to have increased in adults with T2DM (Han and Lin, 2014). Despite conflicting findings, the transplantation of gut microbiota in both animal and human models supported the causal role of gut microbiota in the development of metabolic disorders, including T2DM (Vijay-Kumar et al., 2010; Vrieze et al., 2012).

Gestational diabetes mellitus (GDM) is a transient state of hyperglycemia detected during pregnancy. Profound hormonal, metabolic, and immunological changes occur during pregnancy to sustain the demands of the growing fetus (Mor and Cardenas, 2010). The first trimester is referred to as an anabolic state where maternal insulin secretion and glucose uptake by adipose tissue increases to store an adequate energy supply for fetal development. Thus, pregnant women will start to gain weight. As the pregnancy progresses, the levels of placental and metabolic hormones, as well as pro-inflammatory cytokines, increase and thereby reduce maternal insulin sensitivity during the second half of the pregnancy. During the third trimester, maternal insulin insensitivity stimulates gluconeogenesis and lipolysis, leading to an elevation of maternal plasma glucose and free fatty acids (FFAs) levels. This phase is referred to as a catabolic state in which the maternal plasma glucose and FFAs are transported through the placenta to supply adequate energy for healthy fetal development. However, susceptible pregnant women are unable to compensate for insulin resistance and will develop hyperglycemia due to insufficient insulin secretion secondary to pancreatic β-cell dysfunction (Kim, 2014; Plows et al., 2018).

The Hyperglycemia and Adverse Pregnancy Outcome (HAPO) study involving 15 multinational centers reported the prevalence of GDM between 9.3 and 25.5% in the global population (Sacks et al., 2012). GDM has become a major health burden, as it is associated with poor fetal-maternal outcomes, including polyhydramnios, higher cesarean section rate, pre-eclampsia, shoulder dystocia, and macrosomia (Beucher et al., 2010; Ismail et al., 2011; Catalano et al., 2012; Kc et al., 2015). Furthermore, Ehrlich et al. (2011) reported an increased risk of GDM by 38% in future pregnancies in women diagnosed with GDM during their first pregnancies compared to only 3.5% in women without GDM. In a typical woman with GDM, the hyperglycemic state seen during pregnancy is usually temporary and returns to normal after delivery. However, studies have shown that a proportion of women with a history of GDM develop T2DM. The prevalence of post-GDM women who were diagnosed with T2DM within 5 years was about 20 to 50% (Kim et al., 2002; Eades et al., 2015; Allalou et al., 2016). Moreover, a meta-analysis of 20 studies on postnatal women with a previous history of GDM showed that these women had a 7-fold higher risk of developing T2DM when compared to those with healthy pregnancies (Bellamy et al., 2009). Several factors such as obesity, recurrent GDM, higher glucose levels during pregnancy, and an insulin requirement during pregnancy may influence the risk of persistent postpartum glucose intolerance (Cheung and Helmink, 2006; Kim, 2014). Based on a 5-year prospective study, South Asian women had a significantly higher percentage who developed glucose intolerance compared to post-GDM women from other ethnicities (60%, *p* < 0.05) (Girgis et al., 2012). African-American women have an ~10-fold higher risk of developing T2DM compared to non-Hispanic white women and Asian/Pacific Islanders (hazard ratio 9.9, 95% CI 7.5–13.1) (Kim et al., 2014). Hence, β-cell dysfunction and the risk of T2DM in post-GDM women may be associated with ethnicity. With an increasing incidence of T2DM in post-GDM women, it is necessary to elucidate the pathophysiology of GDM using a new direction.

A recent study has reported alterations of gut microbiota during early pregnancy in obese women were highly associated with a change in metabolic hormones (Gomez-Arango et al., 2016). Elevations of pathobionts (Enterobacteriaceae, *Staphylococcus*, and *E. coli)* and depletions of *Bifidobacterium* and *Bacteroides* were observed in overweight pregnant women compared to normal weight pregnant women (Santacruz et al., 2010). In line with these findings, several studies found gut microbiota to be significantly altered in women with GDM and resembled the gut microbiota profiles of adults with T2DM (Kuang et al., 2017; Crusell et al., 2018; Ferrocino et al., 2018). Thus, gut microbiota could be a potential marker of impaired glucose metabolism during pregnancy. Manipulation of gut microbiota composition may be a promising target to improve health outcomes in women with GDM. Nonetheless, the link between gut microbiota and GDM is still controversial and remains to be elucidated. For instance, a prospective study by DiGiulio et al. (2015) evaluated the microbiota composition of different sites of the body during pregnancy. The gut microbiota constitution and diversity of the vagina, distal gut, saliva, and teeth/gums were relatively stable during pregnancy (DiGiulio et al., 2015). In this review, we discuss gut dysbiosis and diversity in women with GDM, host-gut microbiota interactions, and the roles of probiotics as a potential therapeutic supplement in post-GDM women.

## Gut Microbiota Dysbiosis in Pregnancy and its Association With GDM

Several studies discovered altered gut microbiota composition during pregnancy, and this may be closely related to the pathogenesis of GDM (Kuang et al., 2017; Mokkala et al., 2017a; Crusell et al., 2018; Ferrocino et al., 2018). However, there were studies that reported opposite findings (Koren et al., 2012; Avershina et al., 2014; DiGiulio et al., 2015). A significant remodeling of the gut microbiota composition throughout pregnancy using 16S sequencing was reported by Koren et al. (2012). The gut microbiota composition of pregnant women in the first trimester was comparable to the healthy non-pregnant controls. However, in the third trimester, the relative abundance of Proteobacteria and Actinobacteria significantly increased, while the abundance of *Faecalibacterium* depleted. About 29 distinct operational taxonomic units (OTUs) were identified, of which 18 were over-represented in the first trimester fecal samples, which mainly belonged to butyrate-producing bacteria such as *Faecalibacterium* and *Eubacterium*. The remaining OTUs were over-represented in the third trimester fecal samples, mainly the Enterobacteriaceae family and *Streptococcus* genus. The relative abundance of Bacteroidetes and Firmicutes were, however, maintained throughout the pregnancy. Bacterial richness (α-diversity) was ultimately reduced toward the third trimester (Koren et al., 2012).

In addition, stool energy content significantly increased in the third trimester (Koren et al., 2012). Even though dietary intake was consistent throughout the study, changes in stool energy content observed during the third trimester signifying excess nutrient load may be attributed to the effect of gut microbiota on energy metabolism. However, functional analyses using Kyoto Encyclopedia of Genes and Genomes (KEGG) did not provide evidence of elevation of the metabolic pathway and thus were unable to correlate the effect of gut microbiota dysbiosis on energy metabolism in women with GDM. Fecal samples from the first and third trimesters were pooled from five healthy-weight pregnant women, transplanted into the germ-free wild-type Swiss-Webster female mice, and assessed using shotgun metagenomics analysis. The third trimester recipient mice showed evidence of adiposity and significant elevations of both pro-inflammatory cytokines [e.g., interleukin-1 beta (IL-1β), interleukin-2 (IL-2), interleukin-5 (IL-5), and interleukin-6 (IL-6)] and 30-min postprandial blood glucose levels compared to the first trimester recipient mice. The outcomes were similar to metabolic syndrome phenotypes and consistent with the previous metabolic syndrome mouse model (Vijay-Kumar et al., 2010). In conclusion, the authors discovered aberrant gut microbiota dysbiosis toward the third trimester of pregnancy associated with adiposity, low-grade inflammation, insulin resistance, and hyperglycemia regardless of GDM status (Koren et al., 2012).

In a study involving 75 overweight participants, Mokkala et al. (2017a) published the relationship between gut microbiota dysbiosis in early pregnancy and the onset of GDM. A single fecal sample was obtained during early pregnancy (12.5 weeks of gestation), and the oral glucose tolerance test (OGTT) was measured at 25.5 weeks of gestation. About 15 participants were diagnosed with GDM, and the relative abundance of the Ruminococcaceae family statistically differed between women who developed GDM and women who did not develop GDM. After adjustment for all potential confounding factors, such as pre-pregnancy body mass index (BMI), dietary intake of fat and fiber, and family history of T2DM or metabolic syndrome, a significant association between the Ruminococcaceae family and glucose level was noted with a higher odds ratio for a positive diagnosis of GDM. However, no correlation was detected between the Ruminococcaceae family and insulin and high-sensitivity C-reactive protein (hs-CRP) levels (Mokkala et al., 2017a). Even though it had a small sample size of women who developed GDM, this study had good strength, as it had strictly eliminated likely confounding factors. Overall, this study confirmed the evidence of gut microbiota dysbiosis in early pregnancy and specific microbiota preceding the diagnosis of GDM, suggesting gut microbiota modulation as a potential therapeutic target to prevent GDM. Nevertheless, the link between gut microbiota dysbiosis in women with GDM and inflammation remains to be elucidated.

A study conducted by Kuang et al. (2017) compared the gut microbiota composition between women with GDM and healthy pregnant women between 21 and 29 weeks of gestation. The metagenomics approach using whole metagenome shotgun sequencing demonstrated gut microbiota dysbiosis at the species level in women with GDM compared to women without GDM during the second trimester. Elevations of pathobionts, including *P. distasonis, Klebsiella variicola*, and *Catenibacterium mitsuokai* were observed. While the expression levels of beneficial butyrate-producing bacteria, such as *Alistipess* spp., *Bifidobacterium* spp., *Eubacterium* spp., and *Methanobrevibacter smithii*, were lower compared to the healthy pregnant women. Moreover, metagenomic linkage groups (MLGs), such as GDM67, GDM64, *P. distasonis* [GDM1], *K. variicola* [GMD41], and *E. rectale* [GDM34] positively correlated to maternal glucose levels, further suggesting the association between gut microbiota dysbiosis and glucose intolerance in women with GDM. A MLG is a group of metagenomics material that is physically linked rather than independently distributed (Qin et al., 2012). Furthermore, functional analysis using KEGG has established evidence of gut microbiota dysbiosis, which may interfere with host metabolism in women with GDM. The pathways related to carbohydrate metabolism, such as membrane transport, energy metabolism pathways, lipopolysaccharide, and phosphotransferase system, were enriched in women with GDM, suggesting the possibility of gut microbiota utilizing glucose as a source of energy in women with GDM (Kuang et al., 2017).

A number of reviews have highlighted the link between low-grade inflammation and the development of T2DM (Caricilli and Saad, 2013; Han and Lin, 2014; Tilg and Moschen, 2014). Consistently, few studies have reported altered normal gut microbiota composition in women with GDM that show a positive correlation with adiposity, low-grade inflammation, and glucose intolerance (Cortez et al., 2018; Crusell et al., 2018; Ferrocino et al., 2018). For instance, *Faecalibacterium* was inversely associated with fasting blood sugar (FBS), while OTUs assigned to *Akkermansia* were associated with lower insulin sensitivity. In addition, *Bacteroides* and *Sutterella* were associated with hs-CRP and CRP levels, respectively (Crusell et al., 2018; Ferrocino et al., 2018; Ye et al., 2019). Nonetheless, the association between gut microbiota and inflammation, as well as the risk for GDM, is scarce. Ideally, pro-inflammatory markers, such as IL-1β, IL-6, and tumor necrosis factor-alpha (TNF-α), should be investigated to demonstrate novel insight between gut microbiota dysbiosis and low-grade inflammation in women with GDM.

On the other hand, a study from China explored the relationship between gut microbiota dysbiosis and lipid metabolism in women with GDM using a lipidomics approach (Liu et al., 2019). The relative abundance of *Streptococcus, Veillonella, Prevotella, Haemophilus*, and *Actinomyces* was significantly elevated in women with GDM plus hyperlipidemia cohorts. Furthermore, *Streptococcus, Actinomyces, Veillonella*, and *Haemophilus* were positively correlated to total cholesterol levels. *Prevotella* was significantly correlated with lipid metabolites, such as lysophosphatidylglycerol (LPG) and phosphatidylinositol-3 (PIP3), resembling obese and diabetic phenotypes (Liu et al., 2019). Therefore, these data provide strong evidence that gut microbiota dysbiosis is associated with lipid metabolic mechanisms and may contribute to the pathogenesis of GDM. Wang et al. (2018) investigated the gut microbiota composition of pregnant women and compared it with the microbiota of the oral and vaginal areas. The findings showed the relative abundance of the Firmicutes phylum in the oral microbiota of women with GDM was significantly reduced. However, the gut and vaginal microbiota composition at the phylum level were similar between women with GDM and women without GDM. At the genus level, the gut microbiota of women with GDM was dominated by *Fusobacterium* and the abundance of *Faecalibacterium* was significantly reduced compared to women without GDM (Wang et al., 2018).

A limited number of studies have focused on the gut microbiota of post-GDM women. Reevaluation of gut microbiota at 1 month postpartum showed that gut microbiota dysbiosis and loss of bacteria richness, which occurred during pregnancy, remained persistent following delivery (Koren et al., 2012). Crusell et al. (2018) reevaluated the gut microbiota of 125 participants (43 with GDM and 82 without GDM) at 8 months postpartum. The majority of OTUs associated with post-GDM women was derived from the Actinobacteria and Firmicutes phyla such as *Collinsella, Olsenella*, and *Clostridium*. In contrast, OTUs belonging to *Ruminococcus 2* (OTU_152) (*Lachnospiraceae*), *Oscillibacter* (OTU_371), *Faecalibacterium* (OTU_3232), *Bacteroides* (OTU_4999), and *Isobaculum* (OTU_595) were depleted in post-GDM women (Crusell et al., 2018). Fugmann et al. (2015) investigated the gut microbiota composition of 42 post-GDM women and 35 women without GDM. The women were recruited between 3 and 16 months postpartum. Half of the post-GDM women had persistent postpartum glucose intolerance. At the phylum level, the gut microbiota of the postpartum women were predominantly enriched with Actinobacteria, Bacteroidetes, Firmicutes, Proteobacteria, and Verrucomicrobia phyla regardless of GDM status. In contrast to Crusell et al. (2018), Fugmann et al. (2015) observed the relative abundance of the Firmicutes phylum was significantly lower in post-GDM women compared to women without GDM. At the family level, the proportion of Prevotellaceae was significantly increased in the subgroup of post-GDM women. However, the bacterial richness (α-diversity) was similar between both groups (Fugmann et al., 2015). Our published preliminary data witnessed a similar gut microbiota shift in a group of 12 post-GDM women (Hasain et al., 2019). The relative abundance of gut microbiota composition in post-GDM women with impaired glucose tolerance was enriched with *Prevotella_9*, which was ~18% higher than the post-GDM women with normal glucose tolerance (Hasain et al., 2019). On the other hand, a study by Hasan et al. (2018) on post-GDM women 5 years postpartum showed that there was no significant difference in the gut microbiota composition in post-GDM women compared to healthy women. Interestingly, the *Anaerotruncus* genus was elevated in children of women with GDM, suggesting a possible connection between gut microbiota dysbiosis and GDM (Hasan et al., 2018). These findings are summarized in Table 1.

**Table 1:** GDM-associated gut microbiota of different geographical locations, participant selection, sampling duration, and sequencing methods.

**Publications**	**Location**	**Participants details**	**Time of sampling**	**Sequencing methods**	**GDM associated gut microbiota**
Koren et al., 2012	Finland	15 GDM and 76 non-GDMMostly have normal pre-pregnancy weight	1st trimester, 3rd trimester, 1 month postpartum	16S sequencing, V1, V2 regions	^*^Actinobacteria, Proteobacteria, Enterobacteriaceae, *Streptococcus*
Mokkala et al., 2017a	Finland	15 GDM and 60 non-GDMAge: 18 – 45 y/oAll overweight	± 12.9 weeks of gestation	16S sequencing, region N/A	Ruminococcaceae family
Kuang et al., 2017	China	43 GDM and 81 non-GDMGDM women were older, with higher pre-pregnancy BMI	21–29 weeks of gestation	Whole-metagenome shotgun sequencing	Enterobacteriaceae, *Enterobacter cloacae, Megamonas, Phascolarctobacterium, Parabacteroides distasonis, Klebsiella variicola, E. coli, Coprococcus comes, Catenibacterium mitsuokai, Citrobacter* spp.
Ye et al., 2019	China	24 GDM with good GC12 GDM with failed GC16 non-GDMAverage age and BMI: 35 y/o, 25	24–28 weeks of gestation	16S sequencing, V3–V4 regions	*Blautia, Eubacterium_hallii_*group
Ferrocino et al., 2018	Italy	41 GDMAge: 37.1 ± 4.2BMI: 25.8 ± 5.9Included only Caucasian race	24–28 weeks of gestation, 38 weeks of gestation	16S sequencing, V3–V4 regions	Firmicutes*, Coprococcus, Dorea, Faecalibacterium, L–Ruminococcus, Lachnospiraceae, Collinsella, Bacteroides, Phascolartobacterium, Eryipelotrichia, Sutterella*
Cortez et al., 2018	Brazil	26 GDM and 42 non-GDMWomen with GDM were older (35.07 ± 3.75) and had higher pre- pregnancy BMI (73 vs. 55%).Included White, mixed, and Black ethnicities	3rd trimester	16S sequencing, V4 region	Firmicutes*, Ruminococcus, Eubacterium, Prevotella*
Festa et al., 2018	Italy	10 GDM and 10 non-GDMWomen with GDM were older (36.24 ± 4.4 vs. 32.0 ± 2.7) and had higher BMI (24.6 vs. 22.1)	34–36 weeks of gestation	Ion Torrent Personal Genome Machine	*Bacteroides caccae, massiliensis, thetaiotaomicron*
Liu et al., 2019	China	11 GDM, 11 Hyperlipidemia12 GDM plus hyperlipidemia and11 controlAge range: 27.3 ± 0.6 to 29.3 ± 0.9BMI range: 25.5 ± 0.6 to 26.7 ± 0.6	27–33 weeks of gestation	16S sequencing, V3–V4 regions	*Streptococcus, Veillonella, Faecalibacterium, Prevotella, Haemophilus, Actinomyces*
Wang et al., 2018	China	147 GDM and non-GDM147 fecal samples	1–2 days before delivery	16S sequencing, V3–V4 regions	*Fusobacterium/Faecalibacterium*
Crusell et al., 2018	Denmark	50 GDM and 157 non-GDMAge range 33.3 ± 4.6 to 34.4 ± 4.4BMI range 27.1 ± 4.8 to 29.3 ± 5.6Women with GDM had significantly higher pre-pregnancy BMIIncluded only women with Danish white origin	3rd trimester, 8 months postpartum	16S sequencing, V1–V2 regions	Actinobacteria*, Collinsella, Rothia, Desulfovibrio, Blautia, Faecalibacterium*, Postpartum: Actinobacteria, *Collinsella, Olsenella, Clostridium, Faecalibacterium, Bacteroides, Veillonella, Bavariicoccus, Clostridium sensu stricto, Clostridiaceae_1, Hafnia, Howardella, Dehalobacter*
Fugmann et al., 2015	German	42 post-GDM and 35 non-GDMAge range: 36 (32–38) to 37 (34–39)BMI range: 27.0 (23.9–31.6) to 22.6 (21.3–26.2)Women with GDM had significantly higher BMI	3–16 months postpartum	16S sequencing, V4 region	Bacteroidetes/Firmicutes, Prevotellaceae
Hasain et al., 2019	Malaysia	12 post-GDMPost-GDM women with GI had significantly higher BMI	N/A	16S sequencing, region N/A	Bacteroidetes, Firmicutes, Verrucomicrobia, Proteobacteria, *Prevotella_9*
Hasan et al., 2018	Finland	60 post-GDM, 68 non-GDM, and 109 childrenAge range: 37.7 ± 5.3 to 39.2 ± 4.4BMI range: 30.6 ± 1.8 to 32.9 ± 6.3Mostly advanced age	5 years postpartum	16S sequencing, region N/A	^*^Bacteroides, Faecalibacterium, Subdoligranulum, Lachnospiracea incertae sedis Anaerotruncus genus was elevated in children of women with GDM

*GDM, gestational diabetes mellitus; y/o, years old; BMI, body mass index; GC, glycemic control; N/A, data not documented; GI, glucose intolerance*.

* *Indicates no significant difference between women with and without GDM*.

In summary, studies of women with GDM showed a broad range of gut microbiota dysbiosis, which was associated with several pathobionts derived from Firmicutes, Proteobacteria, Bacteroidetes, and Actinobacteria phyla, including Ruminococcaceae, *Desulfovibrio*, Enterobacteriaceae, *P. distasonis, Prevotella*, and *Collinsella*. On the other hand, beneficial butyrate-producing bacteria, such as *Faecalibacterium* and *Bifidobacterium*, were depleted. Gut microbiota dysbiosis in women with GDM is associated with inflammation, adiposity, and glucose intolerance, which resembles the gut microbiota profile of adults with T2DM. The gut microbiota of those with GDM remained persistent in the postpartum period, suggesting its potential as a predictive biomarker of T2DM. Nevertheless, most of the studies were unable to identify direct causality between the gut microbiota and GDM. Factors influencing the studies on gut microbiota of women with GDM are further discussed in section Factors Influencing the Studies on Gut Microbiota of Women With GDM.

### Factors Influencing the Studies on Gut Microbiota of Women With GDM

Despite having more studies focusing on gut microbiota in women with GDM, data are inconsistent. Several confounding factors contribute to the disparities between the findings, including the study design, geographical locations, sample size, participant selection criteria, gestational age at the time of fecal sample collection, and sequencing methods. For a study design, a prospective observational study is preferable, as it enables determination of causal relationships and, in this case, the relationship between gut microbiota and GDM. However, the majority of the studies employed a cross-sectional study design (Table 1). Moreover, most of the studies were performed in China and Finland, which may introduce data differences in terms of ethnicity and dietary habits. Sample size is an important factor as it may influence the significance of the results and crucial to adopt the findings clinically. The sample size of the studies ranged from 20 to 207 participants during pregnancy and 12 to 125 participants during the postpartum period (Table 1). A few studies had small sample sizes, and some had unequal numbers of subjects in the distribution between the groups with and without GDM (Table 1). The participants were mostly above 35 years old and overweight. Several studies have observed women with GDM were older and had significantly higher BMI compared to women without GDM (Table 1). However, only few studies have documented that they have adjusted these factors to eliminate possible confounders (Mokkala et al., 2017a; Crusell et al., 2018; Ferrocino et al., 2018).

Another important aspect is the timing of the fecal sample collection, which most studies were found to be lacking. The hallmark of gut dysbiosis is reported to occur in the third trimester of pregnancy (Koren et al., 2012). The majority of the studies collected the fecal samples at a single point. Furthermore, only half of the studies collected the fecal samples in the third trimester, whereas several studies collected a single fecal sample in either the first trimester, second trimester or postpartum period only. Indeed, very few studies compared the gut microbiota composition during pregnancy and after delivery (Table 1). Therefore, some of the findings may be inconclusive. The type of sampling could have also been a factor contributing to discrepancies in findings among the studies. Previous evidence has shown that the adherence of pathobionts and some of the host metabolism occurred in the small intestine (Amar et al., 2011; Tremaroli and Bäckhed, 2012). Therefore, sampling of an intestinal specimen is a better option to identify the association between GDM and gut microbiota. However, it may be challenging, as it is an invasive procedure, costly, and time-consuming.

The selection of a suitable sequencing platform is important to facilitate comparison with other studies. The majority of the published studies utilized the 16S sequencing approach using different hypervariable regions (Table 1). The selection of a suitable region, amplicon primer design, and amplification step are very crucial, as it may exert biases and contribute to conflicting results (Jovel et al., 2016; Ranjan et al., 2016). For instance, Crusell et al. (2018) selected V1–V2 regions and used 27F/338R primer for gene amplification, while Wang et al. (2018) amplified the V3–V4 regions with modified 342F and 805R primers. Although the 16S sequencing method is cheaper and efficient, the analysis was confined only to bacteria and archaea. As a result, it was unable to detect the lower taxonomic levels of the gut microbiota composition. An example of a contradicting finding was the high expression level of *Faecalibacterium*, which is known as an anti-inflammatory bacteria, during pregnancy, while it showed a positive correlation with the inflammatory marker hs-CRP (Crusell et al., 2018). The authors hypothesized that the role of *Faecalibacterium* may be strain-specific and suggested that the identification of lower taxonomic levels with shotgun metagenomics sequencing may explain the conflicting results (Crusell et al., 2018).

Only a single study utilized the shotgun metagenomics sequencing approach to identify the gut microbiota composition of women with GDM (Kuang et al., 2017). This study was able to detect gut microbiota dysbiosis in women with GDM at the lower taxonomic levels and found a significant amount of MLGs, which differed between the women with and without GDM. Further analysis using the KEGG pathway was able to explore the functional genes involved. Based on KEGG pathway analyses, certain pathways related to lipopolysaccharide (LPS) biosynthesis and energy metabolism were elevated, and insulin signaling pathways were reduced in women with GDM compared to women without GDM (Kuang et al., 2017; Ferrocino et al., 2018; Ye et al., 2019). On the other hand, Festa et al. (2018) conducted a gut microbiota study in women with GDM and performed molecular sequencing using the Ion Torrent Personal Genome Machine (PGM). This method is part of the second-generation NGS platform where the nucleotide sequences are detected by the changes of the surrounding solution's pH proportional to the number of incorporated nucleotides electronically (Kulski, 2016). The PGM has a flexible workflow and is more affordable compared to other second-generation NGS platforms (Kulski, 2016). However, the results are not comparable to other studies, as most studies used 16S sequencing. The research approach in linking microbiota and their metabolites, as well as the interaction between metabolomics with the host transcriptomes, is still lacking.

Therefore, future large prospective studies with consideration of multiple point fecal samplings, selection of advanced sequencing platforms, and adequate assessment and documentation of confounding factors are warranted to confirm the relationship between gut microbiota and GDM.

## Host-gut Microbiota Interactions

Several hypotheses have been put forward to link gut microbiota dysbiosis with glucose intolerance. Researchers have identified that a high-fat diet (HFD) is associated with modulation of gut microbiota composition and elevation of Gram-negative/Gram-positive bacteria ratio leading to the accumulation of LPSs in plasma. LPS is a major component of the Gram-negative bacterial cell wall. Elevation of LPS is associated with low-grade inflammatory pathways, attenuation of insulin signaling, and glucose intolerance (Cani et al., 2007, 2012; Amar et al., 2011; Bagarolli et al., 2017). Possible mechanisms on how gut dysbiosis influences gut epithelial permeability, colonic LPS concentration, low-grade inflammation, and insulin resistance in women with GDM will be discussed with the evidence from HFD-induced obesity and diabetic mice model. This is the closest model to link gut dysbiosis and women with GDM.

### Gut Microbiota Adherence and Translocation Across the Epithelial Layer of the Gut via Receptors and Phagocytosis

A study associated a HFD with altered gut microbiota based on mice model fed a HFD favoring the adhesion of Gram-negative pathobionts to the intestinal mucosa (Amar et al., 2011). After 2 h of fluorescently labeled, ampicillin-resistant *E. coli* (GFP-*E. coli*) gavage, the number of GFP-*E. coli* showed greater adhesion to the mucosal surface of the duodenum, jejunum, ileum, and cecum in mice fed a HFD compared to the control. Further observation showed the translocation of the GFP-*E. coli* across the epithelial layer of the gut into the mucosa, lamina propria, and submucosa. The total bacteria count in blood, mesenteric adipose tissue (MAT), and mesenteric lymph nodes (MLNs) were increased compared to the control. These findings showed the capability of Gram-negative pathobionts to adhere to the mucosa and translocate across the epithelial layer of the gut. Furthermore, pathobionts are capable of moving effectively across the epithelial layer of the gut with the help of antigen receptors. The bacterial load in blood and MAT was lower in HFD Nod1-knockout mice, CD14-knockout mice and increased in adapter proteins MyD-88 knockout mice. Therefore, it could be concluded that bacterial translocation across the epithelial layer of the gut is dependent on antigen receptors, such as nod-like receptors (Nod1), toll-like receptors (TLR/CD14) and is associated with adapter proteins, myeloid differentiation factor (MyD-88). Besides, the translocation of pathobionts across the epithelial layer of the gut is associated with dendritic cells (DCs). The DCs phagocytose and co-localize the pathobionts from the intestinal lumen across the epithelial layer of the gut and into the systemic circulation. Fluorescently labeled *E. coli* was identified to co-localize with DCs intraluminally and within the enterocytes, lamina propria, and lymph nodes. The DCs were restricted within the lamina propria in mice fed a normal diet (Amar et al., 2011).

### “Leaky Gut” Hypothesis

Goblet cells located within the epithelial layer secrete mucus to ensure an optimal mucosal layer overlying the gut epithelium. Mucosal layer and tight junction proteins within the epithelial layer of the gut, mainly occludin and zonula occludens-1 (ZO-1), play important roles in the host defense against invasion by pathobionts. Apart from binding to antigen receptors and phagocytosis, pathobionts, and LPS can translocate across the epithelial layer of the gut by increasing its permeability. This condition is known as “leaky gut.” Evaluation of intestinal permeability showed marked elevation of both paracellular and transcellular permeabilities and a significant reduction in the cecal length, crypt depth, and number of goblet cells in the HFD model compared to control (Hamilton et al., 2015). Several mechanisms may trigger “leaky gut.” Firstly, pathobionts might induce the condition by disrupting the mucosal layer. *Prevotella* is a mucin-degrading pathobiont found to be elevated in some women with GDM (Wright et al., 2000; Fugmann et al., 2015; Liu et al., 2019). *Prevotella* may impair gut permeability in women with GDM by increasing mucin oligosaccharide degradation beyond the normal limit, causing the thinning of the mucosal layer overlying the epithelial layer of the gut (Wright et al., 2000). Diet-induced obese and diabetic mice models were found to have altered normal gut microbiota composition, which impaired their gut epithelial barrier integrity through downregulation of tight junction protein expression, such as ZO-1 and occludin (Cani et al., 2008; Bagarolli et al., 2017). An intact endocannabinoid (eCB) system strengthens the regulation of gut epithelial permeability via cannabinoid receptors 1 (CB1) and 2 (CB2). This system influences the distribution and localization of tight junction proteins, namely ZO-1 and occludin. Pathobionts impair the tone of the eCB system to induce “leaky gut.” Muccioli et al. (2010) investigated the link between the eCB system and metabolic disorders. Their results showed colonic mRNA expression of CB1 and plasma LPS levels were greatly elevated and associated with adipogenesis in HFD-induced obese and diabetic mice models. In contrast, the application of a CB1 antagonist intraperitoneally in obese mice for 12 days significantly improved gut permeability markers and decreased both plasma LPS levels and obesity (Muccioli et al., 2010). Therefore, the translocation of pathobionts and LPS is closely related to the expression and function of TLR/CD14, Nod1, MyD-88, DCs, and eCB system, and is associated with “leaky gut.”

**Figure 1:**
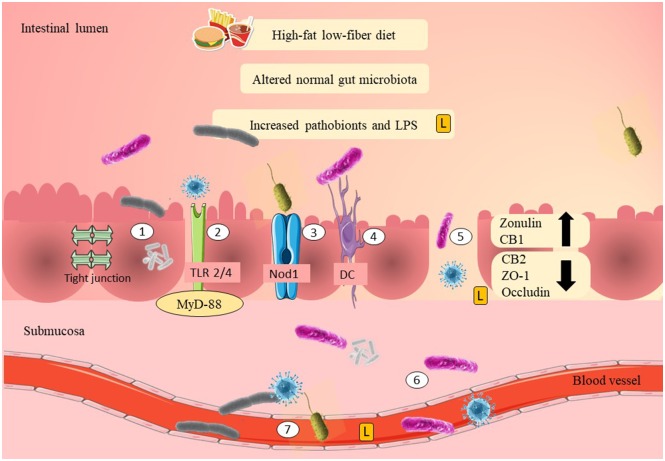
Possible mechanisms of adherence of pathobionts and translocation across the epithelial layer of the gut in GDM. High-fat/low-fiber diet intake might have modulated the normal gut microbiota composition and increased the Gram-negative pathobionts. Elevation of Gram-negative pathobionts might have increased the LPS levels. There are several mechanisms as to how pathobionts and LPS are able to move across the epithelial layer of the gut. The first mechanism is by adherence to the mucosal layer. LPS and pathobionts might have crossed the epithelial layer of the gut through TLR 2/4 activation and is associated with the recruitment of MyD-88. LPS and pathobionts might have crossed the epithelial layer of the gut by binding to Nod1. DCs might have translocated pathobionts by phagocytosis and co-localization of the pathobionts from the intestinal lumen to the systemic circulation. Thin mucosal layer, depletion of tight junction proteins (ZO-1 and occludin), reduction of CB2, and elevation of CB1 may have increased the gut epithelial permeability (i.e., “leaky gut”). “Leaky gut” might have allowed translocation of LPS and pathobionts across the epithelial layer of the gut. LPS and pathobionts might have translocated from the intestinal lumen to the lamina propria and submucosa. LPS and pathobionts might have translocated from the submucosa to the systemic circulation and traveled to the peripheral tissues, including adipose, liver, and skeletal muscle. LPS, lipopolysaccharide; L, lipopolysaccharide; TLR2/4, toll-like receptor 2/4; Nod1, nod-like receptor 1; DC, dendritic cell; CB1/2, cannabinoid receptor 1/2; ZO-1, zona occludens 1; MyD-88, adapter proteins, myeloid differentiation factor.

Zonulin is a physiological modulator of tight junctions and a potential biomarker to predict gut epithelial permeability. Recent evidence has shown that plasma zonulin levels were significantly elevated in women with GDM during their first trimester (Mokkala et al., 2017b; Bawah et al., 2019; Demir et al., 2019). A study in 2019 showed that obese pregnant women with elevated plasma zonulin levels had 109 times more risk of developing GDM compared to women with normal BMI (Bawah et al., 2019). Therefore, this evidence suggests that zonulin may act as a non-invasive biomarker that could potentially link GDM with gut epithelial permeability. The mechanisms of pathobionts and LPS adherence and translocation are summarized in Figure 1.

### Lipopolysaccharides Induced Low-Grade Inflammation and Insulin Resistance

Mice fed a HFD demonstrated a greater elevation of LPS levels (by 2-to 3-fold) and induced metabolic endotoxemia (Cani et al., 2007). LPS contributes to the onset of low-grade inflammation by binding to TLR. Stimulation of TLR recruits the adapter proteins MyD-88 and increases the activities of interleukin-1 receptor-associated kinase (IRAK), transforming growth factor B-associated kinase 1 (TAK1), TNF receptor-associated factor (TRAF6), c-Jun NH2-terminal kinase (JNK) phosphorylation, inhibitory κB kinase-β (IKK- β), and nuclear factor-κb (NF-κB) in mice fed a HFD. Activation of the JNK and IKK-β/NF-κB pathways trigger low-grade inflammation, promote macrophage infiltration, and upregulate pro-inflammatory cytokine mRNA expression, such as IL-1β, IL-6, and TNF-α in the adipose, liver, and muscle tissues. Both the JNK and IKK-β pathways have been linked with serine phosphorylation of the insulin receptor substrate (IRS-1^Ser307^), suppression of phosphatidylinositol 3-kinase (PI3-K), and downregulation of Akt^Ser473^ serine phosphorylation, thereby reducing insulin signaling and impairing glucose uptake in peripheral tissues (Akira and Takeda, 2004; Cani et al., 2007; Kim et al., 2007; Amar et al., 2011; Caricilli and Saad, 2013). These data demonstrated that altered gut microbiota was a possible modulator of insulin resistance mediated by LPS-associated low-grade inflammation.

#### Possible Roles of Gram-Negative Gut Microbiota and Lipopolysaccharides in Women With GDM

**Figure 2:**
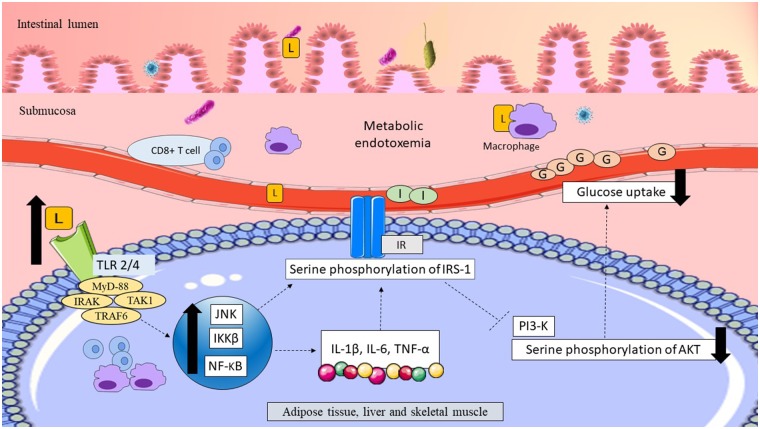
Possible link between LPS and glucose intolerance in women with GDM. Translocation of LPS across the epithelial layer of the gut might have upregulated the pro-inflammatory CD8^+^ T cells and macrophages. Thus, elevation of LPS might have resulted in metabolic endotoxemia. LPS might have traveled to the peripheral tissues, including adipose, liver and skeletal muscle, and become bound to TLR. Activation of TLR might have recruited the adapter proteins MyD-88, IRAK, TAK1, and TRAF6 triggering macrophage infiltration and upregulation of inflammatory pathways (JNK/IKKβ/NF-κB). Upregulation of inflammatory pathways might have elevated pro-inflammatory cytokines, such as IL-1β, IL-6, and TNF-α. Upregulation of JNK/IKKβ/NF-κB might have elevated serine phosphorylation of the IRS-1^Ser307^, causing suppression of PI3-K, and downregulation of Akt^Ser473^. Reduction of Akt^Ser473^ phosphorylation might have impaired insulin signaling and reduced glucose uptake in peripheral tissues, leading to hyperglycemia in women with GDM. LPS, lipopolysaccharide; L, lipopolysaccharide; TLR2/4, toll-like receptor 2/4; adapter proteins MyD-88, myeloid differentiation factor; IRAK, interleukin-1 receptor-associated kinase; TAK1, transforming growth factor B-associated kinase 1; TRAF, TNF receptor-associated factor; JNK, C-Jun N-terminal kinase; IKKβ, inhibitory κB kinase-β; NF- κB, nuclear factor- κB; IL-1β, interleukin-1β; IL-6, interleukin-6; TNF-α, tumor necrosis factor-α; IR, insulin receptor; IRS-1, insulin receptor substrate-1; PI3-K, phosphatidylinositol 3-kinase; AKT, protein kinase B; G, glucose; I, insulin. Dashed lines indicate the possible effects of LPSs.

Women with GDM who consumed a HFD showed a positive association with the Gram-negative pathobiont *Alistipes* (Ferrocino et al., 2018). Several other studies in women with GDM showed consistent elevations of Gram-negative pathobionts, such as *Parabacteroides, E. coli, Prevotella, Sutterella*, and *Desulfovibrio* (Table 1). Accordingly, functional analysis of the gut microbiota revealed a significant increase in pathways related to LPS biosynthesis and its transport system in women with GDM (Kuang et al., 2017; Ferrocino et al., 2018). Elevation of Gram-negative bacteria and LPS in women with GDM might have weakened the gut epithelial permeability and facilitated LPS translocation across the epithelial layer of the gut to the systemic circulation. This phenomenon could have induced metabolic endotoxemia and triggered inflammatory pathways, causing low-grade inflammation. Elevation of low-grade inflammation might have interfered with insulin signaling and regulation of plasma glucose levels in women with GDM, leading to glucose intolerance. The possible link between LPS and glucose intolerance in women with GDM is summarized in Figure 2.

### Roles of Short-Chain Fatty Acids

The second proposed mechanism is through obesity-induced insulin resistance. A study involving 41 overweight women with GDM during their second and third trimesters investigated the possible roles of diet and gut microbiota on energy metabolism during pregnancy (Ferrocino et al., 2018). Dietary recommendations were given to all the participants, and their dietary intakes were assessed at the beginning and at the end of the study period. At the end of the study, most of the participants failed to follow dietary recommendations (non-adherent), and they consumed significantly lower amounts of fiber and higher sugar and fat amounts compared to the group who followed dietary recommendations (adherent). The gut microbiota composition at the end of the study showed the relative abundance of Firmicutes was elevated while the abundance of Actinobacteria and Bacteroidetes were lower in women with GDM (Ferrocino et al., 2018). This “gut microbiota signature” is similar to the phenotype of the metabolic disorder, mainly the obese phenotype (Everard and Cani, 2013). Moreover, inferred metabolic pathways related to carbohydrate metabolism, such as glycolysis/gluconeogenesis, starch and sucrose metabolism, and galactose metabolism were enriched in women with GDM. Also, non-adherent participants were associated with higher weight gain, lipid profile, CRP, and insulin resistance as well as poor glycemic control, suggesting possible connections with diet, gut microbiota, energy metabolism, low-grade inflammation, and insulin resistance in women with GDM (Ferrocino et al., 2018). One of the mechanisms that could explain how diet and gut microbiota may influence obesity and insulin resistance in women with GDM is the role of short-chain fatty acids (SCFAs) in energy metabolism and low-grade inflammation.

#### Roles of Short-Chain Fatty Acids in Energy Metabolism

Human gut lacks enzymes that digest dietary fibers, such as plant cell-wall polysaccharides, oligosaccharides, and resistant starches (Flint et al., 2008). In a healthy population, gut microbiota ferments these non-digestible fibers to produce SCFAs, such as acetate, propionate, and butyrate (Topping and Clifton, 2001). SCFAs play an important role in harvesting extra energy from the undigested diet and in glucose homeostasis (Canfora et al., 2015). The amount and type of SCFAs produced greatly depends on the amount, type of diet, and gut microbiota composition. Generally, the Firmicutes phylum, especially *Faecalibacterium, Roseburia*, and *Bifidobacterium*, produce butyrate, whereas Bacteroidetes produces acetate and propionate (Macfarlane and Macfarlane, 2003). SCFAs modulate host lipid and glucose metabolism through G protein-linked receptors (GPR41 and GPR43) (Tazoe et al., 2008; Blaut, 2015). The majority of butyrate is taken up by the colonocytes as their energy source (Clausen and Mortensen, 1995), while most of the acetate is taken up by the liver and some is metabolized by adipose, muscle, heart, and kidney tissues (Bloemen et al., 2009).

At the adipose tissue, SCFAs promote adipogenesis by high expression of the transcription factor known as peroxisome proliferator-activated receptor γ (PPARγ) (Hong et al., 2005). SCFAs also increase lipid storage capacity by suppressing lipolysis through GPR43 activation, thus reducing FFAs levels in the plasma (Ge et al., 2008). In 3T3-L1 mature adipocytes stimulated with isoproterenol, sodium acetate decreases phosphorylation of hormone-sensitive lipase and causes a significant reduction of FFAs levels in the plasma (Aberdein et al., 2014). Besides, factor-induced adipose factor (FiAF) plays a role in promoting adipogenesis by suppressing adipocyte lipoprotein lipase (LPL). SCFAs have been found to elevate FiAF and to promote adipogenesis (Grootaert et al., 2011). At the liver and skeletal muscle, SCFAs regulate lipid and glucose metabolism through modulation of the AMP/ATP ratio and activation of AMP-activated protein kinase (AMPK). This regulation depends on the type of SCFAs product. Propionate is an essential substrate for gluconeogenesis and downregulates lipogenesis by inhibiting fatty acid synthase (Demigné et al., 1995). In contrast, butyrate and acetate are associated with lipogenesis in the liver (Wolever et al., 1991; den Besten et al., 2013, 2015). Also, SCFAs regulate glucose metabolism by promoting glycogen storage and inhibiting glycolysis in the liver and skeletal muscles (Beauvieux et al., 2008; Fushimi et al., 2018). SCFAs enhance energy expenditure and reduce fat storage in the liver and skeletal muscle by increasing the expression of peroxisome proliferator-activated receptor gamma coactivator (PGC)-1α to trigger fatty acid oxidation (Gao et al., 2009; den Besten et al., 2015). Additionally, SCFAs also activate enteroendocrine L cells, which stimulate glucagon-like peptide-1 (GLP-1) and the gut hormone peptide YY (PYY). GLP-1 promotes insulin secretion and suppresses glucagon release from the pancreas. A study by Zhou et al. (2015) showed high mRNA expression of GLP-1 and PYY were associated with a significant loss of body fat in mice fed with resistant starch. Moreover, the anti-obesity effect was absent in GLP-1 receptor null (GLP-1R KO) mice and in mice that received daily intraperitoneal injections of a PYY receptor antagonist, confirming the causal relationship between GLP-1 and PYY on reduction of body fat accumulation and glucose homeostasis (Zhou et al., 2015).

#### Short-Chain Fatty Acids and Low-Grade Inflammation

SCFA has a potential role in suppressing the inflammatory response (Ohira et al., 2013). It reduced metabolic endotoxemia by significant upregulation of the tight junction protein genes (ZO-1 and occludin) and showed protective effects on gut epithelial permeability (Elamin et al., 2013). Furthermore, the composition of anti-inflammatory regulatory T cells (Treg) was upregulated in the gut and peripheral tissue in mice treated with SCFAs (Furusawa et al., 2013). Adipocytes are the common place where the inflammatory response occurs secondary to the imbalance between anti-inflammatory agents, such as Treg cells, type 1 T helper cells (TH1, CD4^+^), and pro-inflammatory CD8^+^ effector T cells (CD8^+^ T). The elevation of CD8^+^ T cells preceded the macrophage infiltration and adipose tissue inflammation in mice fed a HFD (Nishimura et al., 2009; Nishimura and Nagasaki, 2010). Ohira et al. (2013) showed butyrate administration significantly suppressed the inflammatory activity in the co-cultured macrophages, inhibited lipolytic activity (adipose triglyceride lipase, hormone-sensitive lipase, and fatty acid-binding protein 4) in the co-cultured adipocytes, and attenuated the production of TNF-α and IL-6 in the co-cultured medium. In the human study, diets consisting of SCFAs were found to suppress pro-inflammatory cytokines, such as IL-6 and TNF-α (Roelofsen et al., 2010).

#### Roles of Short-Chain Fatty Acids in Obesity and T2DM

Overall, adequate SCFAs levels are essential to maintain host energy metabolism and inflammatory response. Yet, altered SCFAs levels dysregulate host metabolism and contribute to obesity and T2DM. For example, higher fecal SCFAs levels are associated with altered gut microbiota and adiposity in obese adults compared to lean adults (Schwiertz et al., 2010; Fernandes et al., 2014). Turnbaugh et al. (2006) associated obesity with increased capacity to harvest energy from the diet. Furthermore, the causal role of gut microbiota dysbiosis and obesity was confirmed with a significant increase of body fat in germ-free mice colonized with “obese microbiota” compared to germ-free mice colonized with “lean microbiota” (Turnbaugh et al., 2006). These findings suggested that altered gut microbiota influences dietary fiber fermentation and increases SCFAs production beyond its normal limit, leading to extra energy harvesting capacity and obesity. In contrast, a metagenomic-wide association study showed adults with T2DM had moderate gut dysbiosis and lower levels of butyrate-producing bacteria compared to healthy control (Qin et al., 2012). Functional analysis showed that membrane transport of sugars, branched chain amino acid (BCAA) transport, and oxidative stress response were elevated, while butyrate biosynthesis was reduced (Qin et al., 2012). This study suggested reductions of SCFAs interplay with host energy metabolism and immunomodulation.

#### Possible Roles of Short-Chain Fatty Acids in Energy Metabolism, Inflammation, and Insulin Response in Women With GDM

**Figure 3:**
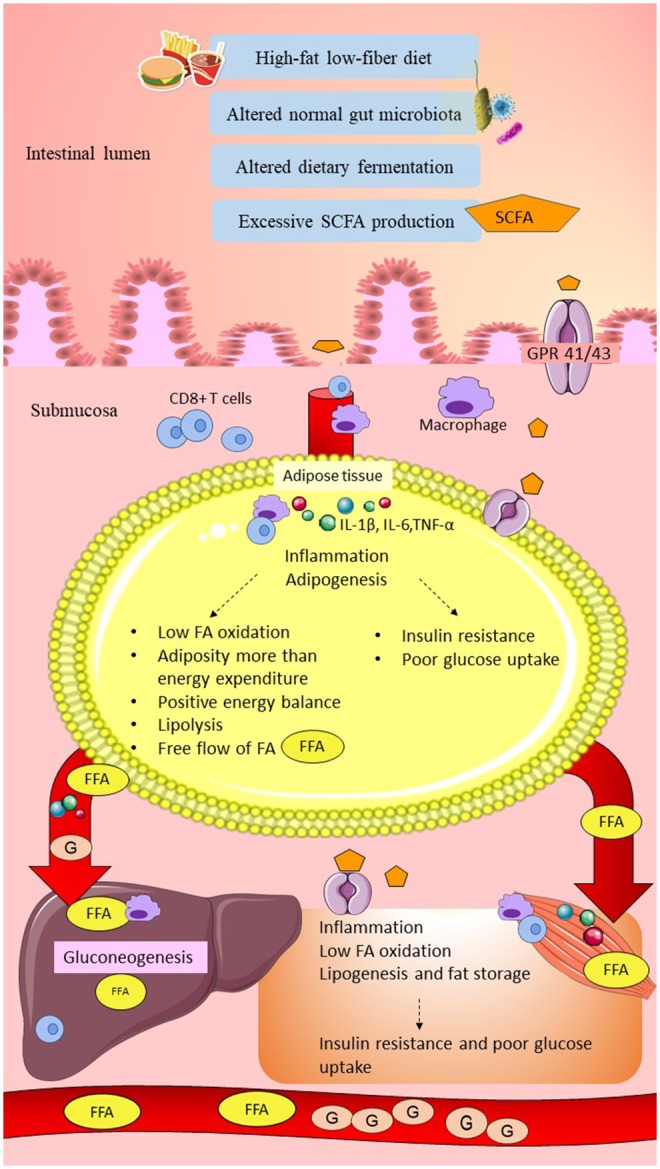
Possible link between SCFAs and glucose intolerance in women with GDM. High-fat/low-fiber diet intake might have altered the normal gut microbiota composition and dietary fermentation. Altered dietary fermentation might have caused excessive SCFAs production and excessive energy harvesting from the diet. SCFAs might have crossed the epithelial layer of the gut through the GPR 41/43 receptor and “leaky gut.” Elevation of SCFAs might have induced metabolic endotoxemia by activation of innate immune cells (CD8^+^ T cells upregulated higher than Treg cells) and macrophage infiltration. SCFAs traveled to the peripheral tissues, including adipose, liver, and skeletal muscle, and might have triggered elevation of pro-inflammatory cytokines, such as IL-1β, IL-6, and TNF-α. In the adipose tissue, excessive SCFAs production might have stimulated adipogenesis beyond adipose tissue storage capacity and higher compared to the energy expenditure (low fatty acid oxidation). Elevation of lipolysis might have caused the overflow of FAs into the systemic circulation. In the liver and skeletal muscle, SCFAs might have increased FFAs uptake and increased lipogenesis, causing fat storage. Elevation of low-grade inflammation and adiposity might have impaired insulin signaling and reduced the glucose uptake, leading to hyperglycemia. Furthermore, excessive SCFAs, especially propionate, might have increased gluconeogenesis in the liver and elevated the plasma glucose levels. In conclusion, low-grade inflammation, insulin resistance, and elevated gluconeogenesis might have caused glucose intolerance in women with GDM. SCFA, short-chain fatty acid; GPR 41/43, G-protein-linked receptor 41/43; Treg cells, regulatory T cells; IL-1β, interleukin-1β; IL-6, interleukin-6; TNF-α, tumor necrosis factor-α; IR, insulin receptor; FA; fatty acid; FFA, free fatty acid; G, glucose. Dashed lines indicate the possible effects of SCFAs.

A high-fat, low-fiber diet in women with GDM might have altered the normal gut microbiota composition, causing elevations of butyrate-producing bacteria such as Firmicutes and *Faecalibacterium*, leading to excessive SCFAs production. Elevations of SCFA might have exceeded the normal lipid storage capacity in the adipose tissue compared to energy expenditure, causing positive energy balance. This might have produced the overflow of FFAs to the systemic circulation, causing an increase in the lipid storage in the liver and skeletal muscle, leading to obesity. Excess SCFAs may promote low-grade inflammation by upregulation of pro-inflammatory markers. SCFAs might have increased glycolysis/gluconeogenesis pathways and inhibited insulin signaling at the peripheral tissues, resulting in hyperglycemia in women with GDM. This hypothesis is consistent with the elevation of carbohydrate metabolism such as glycolysis/gluconeogenesis and reduction of fatty acid metabolism in women with GDM (Ferrocino et al., 2018). The hypothesis linking SCFAs and glucose intolerance in women with GDM is summarized in Figure 3.

On the other hand, several studies observed that the composition of butyrate-producing bacteria, such as *Faecalibacterium* and *Bifidobacterium*, were deficient in women with GDM compared to healthy pregnant women and inversely correlated with glucose tolerance (Kuang et al., 2017; Crusell et al., 2018; Ye et al., 2019). Therefore, a reduction of butyrate-producing bacteria in women with GDM might explain the lower dietary fiber fermentation and reduction of SCFAs production that caused the lower lipid storage capacity in the adipose tissue. Inadequate lipid storage capacity in the adipose tissue, reduction of fatty acid oxidation, and elevation of lipolysis might have elevated the FFAs levels in the circulation, which could have increased the lipid storage in the liver and muscle. Low levels of SCFAs may also be unable to maintain the balance between anti-inflammatory and pro-inflammatory cells. Thus, low levels of SCFAs might have upregulated low-grade inflammation. Obesity and elevation of the inflammatory response might have attenuated insulin signaling and have been associated with hyperglycemia in women with GDM. This hypothesis was consistent with the reduction of several pathways such as the PPAR signaling pathway, adipocytokine signaling pathway, and insulin signaling pathway seen in women with GDM with failure of glycemic control (Ye et al., 2019).

The information on host-gut microbiota interactions in women with GDM is primarily based on previous HFD-induced obesity and diabetic mice models. Moreover, the studies on gut microbiota in women with GDM are based on correlation and thus unable to rule out a causal role between gut microbiota and GDM. Therefore, transplantation of GDM gut microbiota to germ-free mice might be a potential future study to elucidate the actual host-gut microbiota interactions in women with GDM.

## Probiotics as a Potential Preventive Strategy in Women With GDM

Women with a previous history of GDM have a higher risk of developing GDM in future pregnancies and T2DM in later life. Lifestyle modification, including dietary restriction and exercise, are able to modulate gut microbiota composition and improve health outcomes (Wu et al., 2011; O'Sullivan et al., 2015; Ferrocino et al., 2018). Women with GDM who adhered to dietary recommendations had significant reduction of *Bacteroides* and showed better glycemic control (Ferrocino et al., 2018). However, the compliance rate was low among the participants and unsuitable for the long term due to poor health behavior (Kaiser and Razurel, 2013; Kim, 2014; Ferrocino et al., 2018). Pharmacological intervention using metformin after delivery in post-GDM women to prevent T2DM remains controversial, as metformin can have several side effects including abdominal discomfort, diarrhea, dizziness, and hypoglycemia (Buchanan et al., 2012; Stein et al., 2013). Possible host and gut microbiota interactions as explained in the previous sections, may offer a better solution to prevent T2DM in women with GDM through modulation of gut microbiota composition by probiotics supplementation. Probiotics are live microorganisms that give health benefits to the host when administered in adequate amounts (Hill et al., 2014). *Bifidobacterium* and *Lactobacillus* are among the most common, non-pathogenic live microorganisms used as pharmacological interventions (Gomes et al., 2014). Consumption of probiotics requires constant monitoring, as some adverse effects, such as systemic infections, mild gastrointestinal upset, and skin complications, have been reported in ill infants, elderly, immunocompromised individuals, and hospitalized patients (Didari et al., 2014; Sotoudegan et al., 2019). Nevertheless, probiotics exert many favorable health outcomes by increasing good gut microbiota composition, reducing adherence of pathobionts, strengthening gut epithelial permeability, helping in the regulation of immune response, insulin signaling, and energy metabolism. In general, probiotics are safe, well-tolerated, and proven to be beneficial for various diseases, such as metabolic disorders, inflammatory bowel disease, and colorectal cancer (Saez-Lara et al., 2015; Sáez-Lara et al., 2016; Ali et al., 2018; Zaharuddin et al., 2019).

### Roles of Probiotics in Animal Studies

Research on the roles of probiotics in GDM using animal models is lacking. Therefore, possible roles of probiotics obtained from animal studies discussed in this review are related to a HFD- induced obesity and diabetic mice model, as obesity and GDM share similar metabolic disorder phenotypes (Ferrocino et al., 2018). Previous evidence from animal studies has identified several possible mechanisms that could link probiotics and glucose homeostasis.

#### Roles of Probiotics on the Modulation of Gut Microbiota Composition and Reduction of Adherence of Pathobionts

A study on diet-induced obesity (DIO) mice supplemented with probiotics (*Lactobacillus rhamnosus, L. acidophilus*, and *Bifidobacterium bifidumi*) for 5 weeks showed evidence of gut microbiota modulation by significant elevations of both *Bacteroides* and *Alistipes* (Bagarolli et al., 2017). Another study on mice fed a high-fat high-sugar (HFHS) diet showed the proportions of *Adlercreutzia, Clostridium, Streptococcus*, and *Lactobacillus* were decreased in mice supplemented with *L. rhamnosus* Lb102, whereas the abundance of *Bifidobacterium* was greater in mice treated with *B. animalis* ssp. *Lactis* Bf141 (Le Barz et al., 2019). On the other hand, Collado et al. (2007) exhibited the roles of 12 probiotic strains on the reduction of adherence of pathobionts to the intestinal mucosa. Post-probiotics administration, *L. rhamnosus* GG, *L. acidophilus* NCFM, and *B. lactis* Bb12 were among the most adhesive strains with regard to the intestinal mucosa. All probiotic strains significantly reduced and displaced adherence of *Bacteroides, Clostridium, Staphylococcus*, and *Enterobacter* to the intestinal mucosa. Collado et al. (2007) suggested that possible co-aggregation between pathobionts and probiotic strains might have influenced the inhibition or displacement of the adhesion of pathobionts from the intestinal mucosa, but at a different rate depending on the probiotic strain. However, the exact mechanism is not yet fully understood (Collado et al., 2007). Consistently, 1 month of supplementation with *B. animalis* subsp*. Lactis* 420 on a HFD-induced diabetic mice model showed a greater reduction of adherence of *E. coli* in different segments of the mucosa of the small intestine (Amar et al., 2011). Besides, the abundance of *Bifidobacterium* was slightly increased in ileal mucosa (Amar et al., 2011). Therefore, these studies revealed the possible role of probiotics on gut microbiota modulation and inhibition of the adherence of pathobionts to the intestinal mucosa in women with GDM.

#### Roles of Probiotics on Gut Epithelial Permeability

Le Barz et al. (2019) investigated the effects of five different probiotic strains, such as *Lactobacillus* strains (Lb38, *L. plantarum*; L79, *L. paracasei/casei*; and Lb102, *L. rhamnosus*) and *Bifidobacterium* strains (Bf26 and Bf141, two different strains of *B. animalis* ssp. *Lactis*) using a DIO mice model. Probiotics were administered as a single strain to the DIO mice to identify the effects of probiotics on gut epithelial permeability at the strain level. After an 8-week probiotics intervention, the Lb102 probiotics strain significantly upregulated the gene expression of the two important tight junction proteins, namely ZO-1 and occludin. Elevation of gene expression related to mucus production (mucin 2 & 3 and Kruppel-like factor 4) suggested that probiotics may enhance the integrity of the mucosal layer. Significant reduction of CB1 gene expression in HFHS mice treated with *L. rhamnosus* Lb102 and *B. animalis* ssp. *Lactis* Bf141 further supported the role of probiotics on gut epithelial permeability (Le Barz et al., 2019). Similarly, Bagarolli et al. (2017) observed elevations of mRNA expression of both ZO-1 and occludin in the ileum of the DIO mice treated with a combination of *L. rhamnosus, L. acidophilus*, and *B. bifidumi*.

#### Roles of Probiotics on LPS Concentration, Low-Grade Inflammation, and Insulin Signaling

Rodes et al. (2013) reported the use of multiple probiotic treatments (*L. reuteri, L. plantarum, L. rhamnosus, B. animalis, B. longum*, and *B. bifidum*) significantly reduced the concentrations of colonic LPS. *Bacillus longum* subsp*. infantis* showed better capacity to reduce TNF-α concentrations and to increase IL-4 concentrations as compared with other probiotic strains (Rodes et al., 2013). Similarly, 6 weeks of treatment with *B. animalis* subsp. *Lactis* 420 in the HFD-induced diabetic mice model has shown to inhibit Gram-negative bacterial translocation (Amar et al., 2011). The number of Gram-negative bacteria (*E. coli*) in MAT and the expression of pro-inflammatory cytokines, such as IL-1β, IL-6, and TNF-α, were reduced in MAT, liver, and skeletal muscle compared to the untreated mice (Amar et al., 2011). Besides, administration of VSL#3 probiotics consisted of eight probiotic strains (*Streptococcus thermophilus, B. breve, B. longum, B. infantis, L. acidophilus, L. plantarum, L. paracasei*, and *L. delbrueckii* subsp. *Bulgaricus*) in mice fed a HFD downregulated the IKK-β pathway, led to reduction in TNF-α expression and enhanced insulin signaling sensitivity (Ma et al., 2008). Bagarolli et al. (2017) showed the combination of *L. rhamnosus, L. acidophilus*, and *B. bifidumi* administration to DIO mice for 5 weeks reduced TLR protein expression, downstream of JNK and IRS-1^Ser307^ phosphorylation, and recovered the Akt^Ser473^phosphorylation protein expression in the muscle and liver when compared to diet-induced obese mice per fed (DIOPF). Furthermore, DIO mice treated with probiotics showed a reduction of macrophage infiltration in the adipose tissue and better intraperitoneal glucose tolerance test and insulin profile compared to DIOPF mice (Bagarolli et al., 2017). These findings have established the possible roles of probiotics on gut microbiota modulation, reduction of LPS concentration, reduction of bacterial translocation, suppression of low-grade inflammation, and regulation of insulin signaling in women with GDM.

#### Roles of Probiotics on Energy Metabolism

Probiotic supplementation has a beneficial role in host energy metabolism. A study by Yadav et al. (2013) demonstrated significant improvement in adiposity and glucose tolerance in HFD-induced obesity and diabetic mice treated with VSL#3 probiotics. The abundance of *Bacteroides* and *Bifidobacterium*, as well as fecal SCFAs (butyrate) levels, were increased after treatment with VSL#3 probiotics compared to the control. Furthermore, the culture technique system has confirmed the beneficial effect of butyrate on the release of GLP-1 by intestinal L-cells. VSL#3 probiotics also reduced adipocyte size and weight gain (Yadav et al., 2013). Besides, Bagarolli et al. (2017) discovered the administration of the combination of *L. rhamnosus, L. acidophilus*, and *B. bifidumi* to DIO mice was found to increase the expression of FiAF and prevent fat accumulation in the liver parenchyma. Additionally, administration of *L. rhamnosus* Lb102 and *B. animalis* ssp. *Lactis* Bf141 probiotic strains significantly reduced visceral adiposity and improved both insulin sensitivity and glucose tolerance in HFHS-fed mice, suggesting the possible roles of probiotics in energy expenditure and lipid buffering capacity in women with GDM (Le Barz et al., 2019).

### Roles of Probiotics in Human Studies

A limited number of studies have explored the beneficial roles of probiotics in women with GDM. Taylor et al. (2017) published a meta-analysis of four parallel double-blinded randomized control trials (RCTs) involving 288 participants assessing the effects of probiotics in women with GDM. Participants diagnosed with GDM were recruited at 24 to 30 weeks of gestation. All participants were randomly assigned to receive either probiotics or placebo in capsule form for 6 to 8 weeks duration. Two studies reported a significant reduction in FBS as compared to the placebo group (Dolatkhah et al., 2015; Karamali et al., 2016). Although insignificant, the meta-analysis of the four studies showed a reduction of 0.13 mmol/L in FBS compared to the placebo group. Moreover, analysis of insulin resistance using the Homeostatic Model Assessment of Insulin Resistance (HOMA-IR) demonstrated a significant reduction of HOMA-IR by 0.69, suggesting potential roles of probiotics on glucose regulation in women with GDM. However, significant heterogeneity was found across studies (*I*^2^ = 79%, *p* < 0.01) (Taylor et al., 2017). Out of four, only a single study evaluated the effects of probiotics on inflammatory markers (Jafarnejad et al., 2016). Approximately 82 women diagnosed with GDM were randomly assigned to receive either VSL#3 probiotics in a capsule or placebo for 8 weeks. Post-intervention, hs-CRP, TNF-α, and IL-6 levels were significantly reduced in women with GDM who received VSL#3 probiotics compared to the placebo group. This evidence strengthens the possible effect of probiotics on the immune response (Jafarnejad et al., 2016).

Few strengths support the result of the above meta-analysis. All four studies were of high quality, employed a parallel double-blinded randomized control design and were at low risk for bias. Although the duration of the study was short, all four studies were within the same range of 6 to 8 weeks and used similar probiotic preparations (capsules). Treatment with metformin significantly altered the gut microbiota in adults with T2DM, thus the exclusion of participants that require pharmacological intervention is required to eliminate the potential bias (Wu et al., 2017). Almost all of the studies documented the exclusion of participants requiring pharmacological intervention (either metformin or insulin) during the study period. Side effects were not reported in all these studies, and the fetal-maternal outcome was similar compared to the placebo group. This supported that probiotics were generally well-tolerated, safe, and beneficial for women with GDM (Dolatkhah et al., 2015; Lindsay et al., 2015; Jafarnejad et al., 2016; Karamali et al., 2016).

However, there are several differences between the studies that could be a limitation to this meta-analysis. There was a big difference in the sample size, ranging from 60 to 149 participants per study. The probiotics dosage was broad, ranging from 1 billion to 15 billion colony forming units (CFU). Most of the studies used different multi-strain probiotics assigned to *Bifidobacterium* and *Lactobacillus*. However, Lindsay et al. (2015) used a single strain probiotic capsule delivering 1 billion CFU of *Lactobacillus salivarius* UCC118 and did not show an impact on glycemic control. Possible confounding factors include single strain probiotic administration and employment of different glucose tolerance criteria. Lastly, out of the four studies, only Karamali et al. (2016) completely assessed the dietary patterns and physical activity changes throughout the intervention at three points. Other confounding factors that need attention are the duration of probiotics/fermented food/antibiotic consumption before the intervention. Adequate duration is required to enable a sufficient probiotics/fermented food/antibiotics washout period. The duration of the washout period ranged from 10 days to 3 months before the intervention. A study by Jafarnejad et al. (2016) recruited the participants with a 10-day washout period, and this study showed beneficial effects on glucose level, insulin resistance, and inflammatory markers. However, the findings may be inconclusive as the washout period was very short. Finally, another important aspect that was lacking in all four studies was the pre- and post-intervention gut microbiota analyses, which may be a potential predictor to determine the role of probiotics on gut microbiota modulation.

In contrast, a meta-analysis of RCTs on the roles of probiotics in adults with T2DM showed significant evidence of glycemic control, particularly in studies using multi-strain probiotics (Li et al., 2016). FBS was reduced by 0.61 mmol/L, and high-density lipoprotein cholesterol (HDL-C) was increased by 0.42 mmol/L. This meta-analysis showed a greater impact of probiotics on glucose metabolism as it involved 12 RCTs, a larger sample size (714 participants), and the majority of the studies (eight studies) used multi-strain probiotics with a duration of study that ranged from 6 to 12 weeks. Further analysis found that a duration of probiotics of less than 8 weeks showed a better reduction of FBS compared to a duration of probiotics greater than 8 weeks. One of the possible reasons was compliance with the probiotics. A longer duration of probiotics may reduce the compliance rate. Furthermore, significant inter-study heterogeneity was observed in the meta-analysis of FBS and HDL-C. The heterogeneity levels for FBS and HDL-C were 66 and 71% respectively. The composition, dosage, and form of probiotics preparation varied between studies and may have influenced the heterogeneity. Most of the bacteria were derived from *Lactobacillus* and *Bifidobacterium* genera, except for one study that used Brewer's yeast (Hosseinzadeh et al., 2013). The effects of probiotics were found to be strain-specific, but some of the studies did not provide the specific strain of the probiotics used. The dosage of the probiotics ranged from 3 million to 30 billion CFU. The preparation of probiotics included yogurts, tablets, capsules, breads, and sachets (Li et al., 2016).

**Figure 4:**
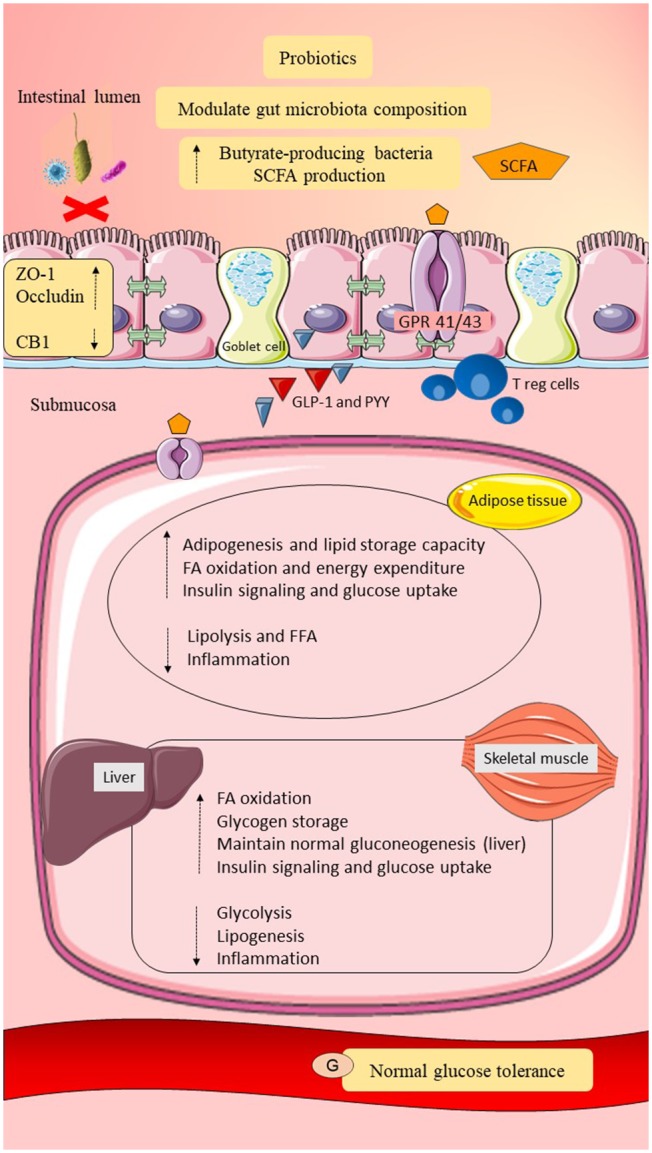
Possible roles of probiotics in post-GDM women. Probiotics may modulate gut microbiota composition by increasing the butyrate-producing gut microbiota and reducing the adherence of pathobionts to the gut epithelial mucosa. Elevation of butyrate-producing bacteria may improve dietary fermentation and promote SCFAs production. Probiotics may improve gut epithelial permeability by upregulating the expression of tight junction proteins (ZO-1 and occludin) and may downregulate CB1. Intact gut epithelial integrity with adequate mucosal layer and tight junctions may reduce pathobionts and LPS translocation and prevent metabolic endotoxemia. Probiotics may upregulate Treg cells and downregulate CD8^+^ T cells. Probiotics may also promote GLP-1 and PYY release. Moreover, probiotics may regulate lipid metabolism by maintaining adipogenesis, fatty acid oxidation, and suppression of lipolysis. Therefore, suppression of metabolic endotoxemia and adiposity, as well as elevation of GLP-1 and PYY, may increase insulin signaling and glucose uptake. Probiotics in an adequate amount may also regulate glucose metabolism by maintaining gluconeogenesis and promoting glycogen storage. In conclusion, probiotics may reduce systemic inflammation and improve lipid and glucose homeostasis in post-GDM women. SCFA, short-chain fatty acid; GPR 41/43, G-protein-linked receptor 41/43; CB1/2, cannabinoid receptor 1/2; ZO-1, zona occludens 1; Treg cells, regulatory T cells; GLP-1, glucagon like peptide-1; PYY, peptide YY; IL-1β, interleukin-1β; IL-6, interleukin-6; TNF-α, tumor necrosis factor-α; IR, insulin receptor; FFA, free fatty acid; G, glucose. Dashed lines indicate the possible effects of probiotics.

Overall, the consumption of multi-strain probiotics consisting of *Lactobacillus* and *Bifidobacterium* may enhance health outcomes in women with GDM by modulating gut microbiota composition, reducing adherence of pathobionts, improving gut epithelial integrity, suppressing the inflammatory response, regulating lipid metabolism, enhancing insulin signaling, and alleviating glucose intolerance. The selection of a suitable probiotics strain is essential, as the effects of probiotics are influenced by the type of probiotics strain used. The possible roles of probiotics in post-GDM women are summarized in Figure 4. Human fecal transplantation into the mice may highlight the molecular mechanisms on how probiotics act in women with GDM. Moreover, the gut microbiota profiling during interventions with probiotics may strengthen the roles of probiotics on gut microbiota modulation. Nevertheless, available data on the roles of probiotics are inconsistent and may be due to several factors, such as small sample size, short duration of intervention, confounding factors, and probiotics selection. Besides, conclusion on standardized probiotics selection, recommended dosage, and duration of probiotics supplementation require further investigation.

## Conclusion

Gut microbiota dysbiosis during pregnancy may contribute to the pathogenesis of GDM and risk of T2DM in post-GDM women, suggesting gut microbiota is a predictive biomarker of T2DM. Knowledge on the possible host-gut microbiota interactions in GDM could offer a potential therapeutic target to improve health outcomes in women with GDM. Dysbiosis was linked with adiposity, low-grade inflammation, insulin resistance, and hyperglycemia. Although lifestyle modification (diet and exercise) may prevent or delay the onset of T2DM in women with GDM, it was inefficient. Probiotics supplementation, especially multi-strain probiotics from *Bifidobacterium* and *Lactobacillus*, was found to modulate gut microbiota composition, reduce LPS levels, maintain SCFAs concentrations, and enhance health outcomes. Therefore, a synergistic approach involving both lifestyle modification (exercise and diet) and probiotics supplementation could be a novel approach to prevent glucose intolerance in women with GDM.

To date, the findings on gut microbiota in women with GDM is limited, inconclusive, and conflicting, but this could be a good start to further explore the roles of gut microbiota in women with GDM. Indeed, the roles of gut microbiota to host metabolism are postulated as strain-specific. Therefore, integration of a shotgun metagenomics approach using standardized DNA extraction and combination with a multi-omics approach, such as transcriptomics, proteomics or metabolomics, may enable better identification of the gut microbiota at the strain level and to fully elucidate the potential roles of gut microbiota in women with GDM. Moreover, the benefits of probiotics need to be considered wisely and carefully monitored, as most of the evidence was derived from animal studies, and supplementation of unknown pathogenic bacteria may cause side effects in susceptible individuals. Thus, long-term prospective and interventional studies using multi-strain probiotics involving large post-GDM cohorts from various ethnicities with consideration of possible confounding factors, such as age, BMI, diet, physical activities, and drugs are warranted.

## Author Contributions

RR and NMM were the the principal investigators and were responsible for the original ideas of the project. ZH and JG drafted and edited the manuscript. NR gave advice on the nutritional aspect of the study. NK was involved in the study as endocrinologist. NAM as obstetrician, gave advice on the study.

## Conflict of Interest

The authors declare that the research was conducted in the absence of any commercial or financial relationships that could be construed as a potential conflict of interest.
